# Translations of the Humeral Head Elicit Reflexes in Rotator Cuff Muscles That Are Larger Than Those in the Primary Shoulder Movers

**DOI:** 10.3389/fnint.2021.796472

**Published:** 2022-02-02

**Authors:** Constantine P. Nicolozakes, Margaret S. Coats-Thomas, Daniel Ludvig, Amee L. Seitz, Eric J. Perreault

**Affiliations:** ^1^Department of Biomedical Engineering, Northwestern University, Evanston, IL, United States; ^2^Shirley Ryan AbilityLab, Chicago, IL, United States; ^3^Feinberg School of Medicine, Northwestern University, Chicago, IL, United States; ^4^Department of Physical Therapy & Human Movement Sciences, Northwestern University, Chicago, IL, United States; ^5^Department of Physical Medicine and Rehabilitation, Northwestern University, Chicago, IL, United States

**Keywords:** stretch reflex, glenohumeral joint, glenohumeral stability, reflex amplitude, reflex latency, reflex gain-scaling, fine-wire intramuscular electromyography, surface electromyography

## Abstract

Muscle activation helps stabilize the glenohumeral joint and prevent dislocations, which are more common at the shoulder than at any other human joint. Feedforward control of shoulder muscles is important for protecting the glenohumeral joint from harm caused by anticipated external perturbations. However, dislocations are frequently caused by unexpected perturbations for which feedback control is essential. Stretch-evoked reflexes elicited by translations of the glenohumeral joint may therefore be an important mechanism for maintaining joint integrity, yet little is known about them. Specifically, reflexes elicited by glenohumeral translations have only been studied under passive conditions, and there have been no investigations of how responses are coordinated across the functional groupings of muscles found at the shoulder. Our objective was to characterize stretch-evoked reflexes elicited by translations of the glenohumeral joint while shoulder muscles are active. We aimed to determine how these responses differ between the rotator cuff muscles, which are essential for maintaining glenohumeral stability, and the primary shoulder movers, which are essential for the large mobility of this joint. We evoked reflexes using anterior and posterior translations of the humeral head while participants produced voluntary isometric torque in six directions spanning the three rotational degrees-of-freedom about the shoulder. Electromyograms were used to measure the stretch-evoked reflexes elicited in nine shoulder muscles. We found that reflex amplitudes were larger in the rotator cuff muscles than in the primary shoulder movers, in part due to increased background activation during torque generation but more so due to an increased scaling of reflex responses with background activation. The reflexes we observed likely arose from the diversity of proprioceptors within the muscles and in the passive structures surrounding the shoulder. The large reflexes observed in the rotator cuff muscles suggest that feedback control of the rotator cuff augments the feedforward control that serves to compress the humeral head into the glenoid. This coordination may serve to stabilize the shoulder rapidly when preparing for and responding to unexpected disturbances.

## Introduction

The shoulder is the most mobile joint in the human body (Boone and Azen, 1979), allowing for the completion of complex functional and recreational activities. The anatomy of the glenohumeral joint, with few passive constraints on joint rotation (Soslowsky et al., 1992), facilitates the shoulder’s expansive mobility but increases its susceptibility to instability, which is defined clinically as pain and discomfort due to excessive humeral head translation (Lippitt et al., 1991). The most severe consequence of instability is dislocation or translation of the entire humeral head beyond the rim of the glenoid fossa. Dislocations occur more commonly in the shoulder than in any other joint (Kerr et al., 2011). To maintain glenohumeral stability without compromising mobility, the muscles crossing the glenohumeral joint must provide active stability, which is achieved through a combination of voluntary feedforward control and involuntary feedback control (Labriola et al., 2005; Veeger and Van Der Helm, 2007). Feedforward control is particularly important for protecting the shoulder in response to predictable external perturbations that can cause humeral head translations. Shoulder dislocations, however, are more commonly caused by unexpected perturbations (Longo et al., 2011; Montgomery et al., 2019), for which feedback control is essential.

Stretch-evoked reflexes are a commonly studied form of feedback control that have been shown to increase the stiffness of several other joints (Sinkjaer and Hayashi, 1989; Carter et al., 1990; Kearney et al., 1997). A few studies have characterized stretch-evoked reflexes at the shoulder in response to rotations of the glenohumeral joint. These have demonstrated differences in the characteristics of reflex amplitudes and latencies between rotator cuff muscles and other muscles that primarily move the shoulder (Myers et al., 2003, 2004; Day et al., 2012; Nicolozakes, 2021). However, the feedback control most relevant to preventing shoulder dislocations is that which responds to unexpected translations, not rotations, of the shoulder. Translations of the humeral head will excite the varied proprioceptors in the ligaments and capsule surrounding the shoulder (Guanche et al., 1999; Witherspoon et al., 2014) as well as the muscle spindles most commonly associated with stretch-evoked reflexes. These translations elicit reflexively induced changes in muscle activation but have only been assessed during passive conditions (Latimer et al., 1998) that are less relevant to the functional states in which dislocations typically occur.

Rotator cuff muscles are believed to function as the primary stabilizers of the glenohumeral joint, complementing the role of primary shoulder movers that generate the torques required to move the shoulder through its range of motion. Rotator cuff muscles are regarded as active stabilizers because they have lines of action that pull the humeral head into the glenoid fossa (Lee et al., 2000), stabilizing the joint when activated voluntarily (Lippitt and Matsen, 1993; Lippitt et al., 1993). In contrast, the primary shoulder movers have more anterior or posterior lines of action that can destabilize the joint if agonists and antagonists are not activated in coordination (Ackland and Pandy, 2009). If rotator cuff muscles contribute to feedback stability, it would be expected that they would exhibit large reflexes in response to translational perturbations. However, their lines of action are likely to result in smaller muscle length changes in response to glenohumeral translations than would occur in the primary shoulder movers. It, therefore, remains unclear if the feedback control of the muscles crossing the shoulder is structured to leverage the anatomical differences between the rotator cuff muscles and the primary shoulder movers.

The purpose of this study was to compare stretch-evoked reflexes elicited by translations of the glenohumeral joint between rotator cuff muscles and primary shoulder movers. We hypothesized that reflexes would be larger in the rotator cuff muscles than in the primary shoulder movers based on the different functional roles of these groups. To test our hypothesis, we elicited reflexes by translating the humeral head anteriorly and posteriorly within the glenohumeral joint. Reflexes were recorded using electromyograms while participants produced isometric shoulder torques in multiple directions and at multiple levels of exertion to create a diverse set of active conditions that reflect daily shoulder use. Our results suggest that feedback control of the shoulder is organized to exploit the anatomical arrangement of muscles crossing the shoulder so as to protect against dislocations due to externally imposed translations.

## Methods

### Overview

Our primary experiment was designed to elicit reflexes in shoulder muscles with translational perturbations, allowing us to compare the characteristics of these reflexes between rotator cuff muscles and primary shoulder movers. The deep rotator cuff muscles required fine-wire intramuscular electromyogram (EMG) recordings, whereas surface EMG recordings were made in all other muscles. We, therefore, conducted a secondary control experiment to determine if these different recording modalities influenced our measures of EMG, and in particular the latencies and amplitudes of the measured reflexes.

### Participants

Fifteen healthy adults (eight female, seven male; mean age ± SD: 25.5 ± 4.2 years) participated in the primary experiment of this study. Seven healthy adults (three female, four male; mean age ± SD: 29.1 ± 5.8 years) participated in the control experiment. All participants reported no history of shoulder injury or shoulder pain in the 6 months prior to testing that prevented participation in overhead activities or required treatment from an allied health professional. All participants were right-hand dominant to accommodate for testing of the dominant arm in our robotic system. Participants gave written informed consent prior to the experiment. All procedures and protocols were approved by Northwestern University’s Institutional Review Board (STU00208382).

### Equipment

**Figure 1:**
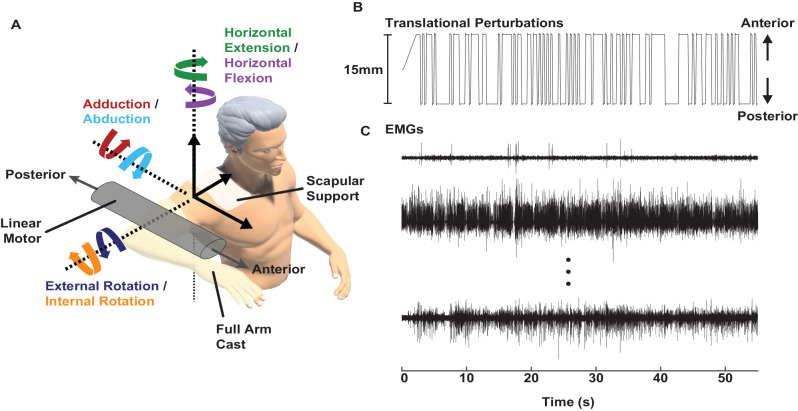
Experimental setup. **(A)** A linear motor applied anterior-posterior perturbations to the glenohumeral joint while participants produced multiple levels of submaximal isometric torque in six directions. **(B)** Perturbations with a pseudorandom binary sequence and 15 mm peak-to-peak amplitude were applied to the glenohumeral joint. **(C)** Electromyograms (EMGs) were recorded in nine shoulder muscles while the participants held isometric torque and the linear motor applied perturbations. Representative samples are shown from the *supraspinatus, infraspinatus*, and *pectoralis major* (top to bottom).

Stretch-evoked reflexes were elicited using a computer-controlled, single-degree-of-freedom linear motor (ThrustTube, Copley Controls Corporation; Canton, MA) with methods adapted from a prior protocol designed to estimate glenohumeral joint mechanics (Nicolozakes et al., 2021). Each participant was seated in a Biodex chair (Biodex Medical Systems; Shirley, NY). The right arm was attached midway between the acromion and the olecranon to the linear motor via a custom-made full-arm fiberglass cast. The upper arm was positioned at 90° shoulder abduction, 20° horizontal flexion, and 0° rotation (Figure 1). The elbow was held at 90° flexion, setting the forearm in the transverse plane. Each participant’s scapula was stabilized with a form-fitting thermoplastic clamp over the acromion and posterior scapula to limit scapulothoracic movement and isolate displacements to the glenohumeral joint. The linear motor applied anterior-posterior displacements to translate the humerus within the glenoid fossa. The full-arm cast helped distribute the forces applied by the linear motor across the entire upper arm to minimize rotations at the glenohumeral joint. The motor was instrumented with a linear encoder (RGH24, Renishaw; Gloucestershire, UK) to record the displacements. Both mechanical and electrical safety stops were used to limit displacements within a safe range. Control of the linear motor was performed using xPC Target (MathWorks; Natick, MA).

EMGs were recorded to quantify the background activity and stretch-evoked reflexes elicited in each of the nine recorded shoulder muscles (Table 1). In our primary experiment, EMGs were recorded from nine total muscles. Bipolar surface electrodes (Delsys Inc.; Natick, MA) were used to record activity from six superficial muscles following standard skin preparation (Merletti and Cerone, 2020): *deltoid* (anterior, middle, and posterior heads), *pectoralis major* (clavicular head), *latissimus dorsi*, and *teres major*. These six muscles predominantly generate torque to rotate the shoulder when active, and we describe them throughout as primary shoulder movers. Fine-wire intramuscular electrodes (Motion Lab Systems; Baton Rouge, LA) were used to record activity from three deep rotator cuff muscles that are difficult to study with surface electrodes (Waite et al., 2010): *supraspinatus*, *infraspinatus*, and *subscapularis*. The rotator cuff muscles generate torque at the shoulder, but more importantly increase the stability of the glenohumeral joint when active by compressing the humeral head into the glenoid (Lee et al., 2000). The broad actions of each primary shoulder mover and rotator cuff muscle are summarized in Table 1.

**Table 1:** Muscles recorded by EMG and their corresponding electrode placements.

	Muscle	Placement	Orientation	Muscle Actions at the Shoulder^d^
Intramuscular Electrodes	*Supraspinatus m*.	Two finger widths superior to the scapular spine at the midpoint between the posterior acromion angle and trigonum spinae.^a^	N/A	Abduction
	*Infraspinatus m*.	Two finger widths inferior to the scapular spine at the midpoint between the posterior acromion angle and trigonum spinae.^a^	N/A	External Rotation
	*Subscapularis m*.	Three finger widths anterior to the midpoint between the inferior angle of the scapula and the anterior axillary fold.^c^	N/A	Adduction, Internal Rotation
Surface Electrodes	*Anterior deltoid m*.	One finger width distal and anterior to the acromion.^b^	Along the line between the acromion and thumb.	Internal Rotation, Horizontal Flexion
	*Medial deltoid m*.	Over the greatest bulge in the muscle between the acromion and lateral epicondyle.^b^	Along the line between the acromion and the lateral epicondyle.	Abduction
	*Posterior deltoid m*.	Two finger widths posterior to the angle of the acromion.^b^	Along the line between the acromion and the little finger.	External Rotation, Horizontal Extension
	*Pectoralis major m*. (clavicular head)	Two finger widths inferior to the midpoint of the clavicle.^a^	Along the line between the sternoclavicular joint and anterior axillary fold.	Adduction, Internal Rotation
	*Latissimus dorsi m*.	Three finger widths distal to the posterior axillary fold.^a^	Along the line between the posterior axillary fold and L3 vertebra.	Adduction, Internal Rotation, Horizontal Extension
	*Teres major m*.	Three finger widths superior to the inferior angle of the scapula along the lateral border of the scapula.^a^	Along the line between the posterior axillary fold and the inferior angle of the scapula.	Adduction, Internal Rotation

*^a^Perotto and Delagi (2011); ^b^Hermens et al. (2000); ^c^Nemeth et al. (1990); ^d^Agur and Grant (2013)*.

In our control experiment, EMGs were recorded from the *anterior deltoid*, *posterior deltoid*, and *latissimus dorsi*. Surface electrodes and fine-wire intramuscular electrodes were used to simultaneously record activity in each muscle. Surface electrodes were placed as described in Table 1. Fine-wire intramuscular electrodes were inserted approximately 5 mm to the side of the surface electrode and angled so that the bare tips of the fine wires were below the middle of the surface electrode to ensure both electrodes recorded from the same area of muscle (Semciw et al., 2014).

Raw EMG signals were recorded using a Delsys Bagnoli-16 EMG system (Delsys Inc.; Natick, MA) and band-pass filtered by the EMG system at 20–450 Hz (surface electrodes) or 20–2,000 Hz (intramuscular electrodes). The gain for all EMG channels was set at 1 K unless a gain of 10 K was required to maximize the range of the data acquisition system. EMG data were then sampled at 5,000 Hz (PCI-6289 data acquisition card, National Instruments; Austin, TX).

### Experimental Protocol

Prior to recording stretch-evoked reflexes, all participants produced isometric maximum voluntary contractions (MVC) in six directions (abduction/adduction, internal/external rotation, and horizontal flexion/extension) while in the experimental setup (Figure 1; Besomi et al., 2020). The torques measured during MVCs were used to normalize torque production in subsequent trials.

Stretch-evoked reflexes were elicited by applying small, stochastic, anterior-posterior perturbations at the glenohumeral joint with the linear motor. The perturbations were generated using a pseudorandom binary sequence. Individual perturbations had a peak-to-peak amplitude of 15 mm, a 100 ms long ramp, and a minimum switching time of 300 ms before the next perturbation (Figure 1). A 15 mm amplitude was large enough to elicit reflexes in all recorded shoulder muscles but small enough to ensure glenohumeral translations were safe for all participants. The midpoint of the perturbations coincided with the humeral head being centered in the glenoid fossa.

The experiment was designed to elicit stretch-evoked reflexes while participants produced volitional torque in different directions and at different levels of effort. Participants generated torque in six different directions to allow for the characterization of reflexes during different combinations of shoulder muscle activity. During each trial, participants maintained a constant isometric torque of either 5% or 10% MVC in one of six directions. The chosen torque levels produced muscle activations large enough to observe consistent stretch-evoked reflexes but without muscle fatigue. Passive trials, during which the participants were instructed to relax and ignore the perturbations (0% MVC), were also recorded. Participants were aided by visual feedback to assist with acquiring and maintaining the target torque for each trial. They were instructed to maintain the target torque on average and to not respond to individual perturbations to minimize the influence of voluntary intervention (Shemmell et al., 2009). The order of torque magnitudes and directions was randomized for each participant. Each trial lasted 55 s. Data from the first 5 s of each trial were discarded to eliminate transient behaviors associated with the onset of the perturbation. Three trials were completed for each condition, resulting in 39 trials per participant (6 torque directions × 2 torque levels × 3 repetitions = 36 active trials + 3 passive trials). Participants rested for a minimum of 10 s between trials to prevent fatigue.

### EMG Processing

EMG signals were used to measure background muscle activity prior to the onset of each perturbation and the reflexively elicited changes in muscle activation in response to each perturbation. All raw EMG signals were detrended, notch filtered at 60 Hz, and digitally band-pass filtered between 20 Hz and 500 Hz (surface) or 60 Hz and 1,500 Hz (intramuscular) with a 4th order Butterworth filter. We performed forward and backward digital filtering to avoid phase shifts. A high-pass cutoff of 60 Hz was used for intramuscular electrodes to reduce motion artifact created by the vibration of the fine-wires from the rapid perturbations. EMG data were rectified prior to any further processing. Rectified EMGs for each muscle were then normalized to the mean rectified value produced during MVCs at the beginning of the experiment. EMGs affected by uncommon or excessive noise during set up or testing were removed prior to analysis. Across the nine muscles recorded from each of our fifteen participants in our primary experiment, nine recordings were eliminated: 1 × *supraspinatus*, 2 × *infraspinatus*, 3 × *subscapularis*, 1 × *pectoralis major*, 1 × *latissimus dorsi*, and 1 × *teres major*. Across the three muscles recorded from each of our seven participants in our control experiment, four recordings were eliminated: 2 × *anterior deltoid* and 2 × *latissimus dorsi*.

**Figure 2:**
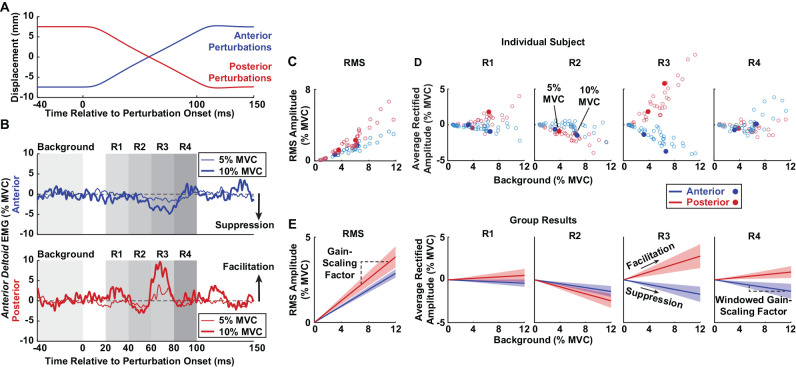
Methods for estimating stretch-evoked reflexes. Sample data are displayed from a single participant to illustrate the methodology. **(A)** Anterior (blue) and posterior (red) perturbations were applied to the glenohumeral joint. Each perturbation had a peak-to-peak amplitude of 15 mm. **(B)** Average rectified EMGs of the *anterior deltoid* are displayed following anterior (top) and posterior (bottom) perturbations as participants generated torque at 5% (thin traces) and 10% (thick traces) MVC. Rectified EMGs were averaged across all perturbations in a trial and plotted relative to the average background EMG (0–40 ms prior to perturbation onset). RMS amplitudes were computed from the root mean square (RMS) of the reflex responses between 20 and 100 ms in each perturbation direction. Average rectified amplitudes in individual time windows (R1–4) were estimated by averaging the rectified EMG activity in four time windows post-perturbation onset. Reflexes that increased compared to background represented facilitation, and reflexes that decreased compared to background represented suppression. **(C)** RMS amplitudes of the *anterior deltoid* from all trials in an individual participant are plotted based on background activity, with the filled dots corresponding to the trials shown in panel **(B)**. **(D)** Average rectified amplitudes of the *anterior deltoid* from all trials in an individual participant are plotted for each reflex window based on background activity. The filled dots correspond to the trials shown in panel **(B)**. **(E)** The group results are displayed for *anterior deltoid* reflexes. The slopes between the RMS amplitudes and background activity were defined as the gain-scaling factors. The slope between the reflex responses in each window and background activity was defined as the windowed gain-scaling factor. Shaded regions represent the confidence intervals of the gain-scaling factors.

EMG recordings from each trial were segmented and aligned to the onset of each anterior or posterior perturbation within the pseudorandom binary sequence. Each trial contained approximately 40 perturbations in each direction. Data between 40 ms prior to the perturbation onset and 100 ms after the perturbation onset were analyzed (Figures 2A,B). EMG after 100 ms were not considered to minimize contributions from voluntary interventions and the cessation of the ramp portion of the perturbation (Lewis et al., 2005; Honeycutt and Perreault, 2012). The aligned segments were averaged before further analysis. Background muscle activity was calculated as the mean average rectified EMG 0–40 ms prior to the onset of the perturbation. All reflex responses were measured relative to this background. We estimated reflex latencies for all active trials as the time after perturbation onset when the average rectified EMG diverged positively or negatively from the background by at least two standard deviations; these results were confirmed visually. Latencies were not measured for passive trials, which had inconsistent reflex responses.

We characterized the reflex amplitude in two ways. First, to capture the net change in muscle activity that resulted from perturbations in each direction, we computed the root mean square (RMS) amplitude as the RMS of the average rectified EMG relative to the background activity. This measure was computed over the period from 20 to 100 ms after perturbation onset. Second, we quantified the average rectified amplitude relative to the background activity in four separate time windows following perturbation onset to assess the sign (facilitatory or suppressive) and time evolution of the observed responses, as done in numerous other studies (Nakazawa et al., 1997; Pruszynski et al., 2009; Shemmell et al., 2009). The windows were: R1 (20–40 ms), R2 (40–60 ms), R3 (60–80 ms), and R4 (80–100 ms; Figure 2B). The R1 window likely included predominantly spinal contributions, and R2 to R4 likely contained contributions from both spinal and supraspinal pathways (Lewis et al., 2004; Shemmell et al., 2009).

### Statistical Analysis

Our primary hypothesis was that the amplitudes of stretch-evoked reflexes in the rotator cuff muscles would be larger than those in the primary shoulder movers. This was assessed grossly by examining the RMS reflex amplitudes across all trials conducted at the largest tested voluntary contraction level of 10% MVC when reflexes were expected to be largest. The distribution of reflexes within each muscle from each participant was summarized by the 10th, 50th, and 90th percentiles to represent small, median, and large responses (Jonsson, 1978; Ludvig et al., 2019). This approach allowed for the comparison of reflex magnitudes beyond the simple mean or median of the data and accounted for the skewed distributions within each muscle and muscle group. A linear mixed effects model was used to determine if there were differences across muscle groups at each percentile. RMS amplitude was considered as the dependent variable. Muscle group and percentile were the fixed independent factors. Participants were treated as a random factor. *Post hoc* comparisons were used to compare amplitudes from the rotator cuff muscles to those in the primary shoulder movers.

Since reflexes scale with background activity (Matthews, 1986), we conducted two additional analyses to determine why reflex amplitudes may differ between rotator cuff muscles and primary shoulder movers. First, we compared the background activity between groups at 10% MVC using an analysis identical to that described for the RMS amplitudes. Next, we examined if the sensitivity of reflex amplitudes to background activity differed between muscle groups by computing a gain-scaling factor for each muscle, defined as the slope between background activity and RMS reflex amplitude. Reflex amplitudes from trials at contraction levels of 0%, 5%, and 10% MVC were included to maximize the range of each muscle’s background activity. Separate gain-scaling factors were computed for each perturbation direction (Figures 2C–E). We compared gain-scaling factors in each muscle group using a linear mixed effects model with RMS reflex amplitude as the dependent variable, background activity as a continuous factor, and muscle and perturbation direction as fixed factors. Participants were treated as a random factor for both intercepts and slopes. The gain-scaling factors for each muscle group were compared using *post-hoc* comparisons. We compared gain-scaling factors between muscle groups separately for each perturbation direction and also stratified by perturbation directions that elicited facilitative or suppressive responses.

Our secondary hypothesis was that the latencies of stretch-evoked reflexes would differ in rotator cuff muscles compared to primary shoulder movers. We used a linear mixed effects model to quantify the average reflex latency for each muscle in each perturbation direction. Participants were treated as a random factor. We tested our hypothesis by comparing the average reflex latencies of the rotator cuff muscles to those of the primary shoulder movers.

In our control experiment, we compared reflexes recorded in the same muscle with surface and fine-wire intramuscular electrodes. First, we used a linear mixed effects model to determine if there were differences in RMS reflex amplitudes between electrode types at the 10th, 50th, and 90th percentiles. Second, we computed gain-scaling factors and compared them between electrode types using a linear mixed effects model. Finally, we used a linear mixed effects model to compare the average reflex latency between electrode types in each perturbation direction. All models included the same fixed, continuous, and random factors described above.

To compare parameters within each linear mixed effects model, we used the Wald t-test statistic with a Satterthwaite approximation to estimate P-values (Luke, 2017). All confidence limits reported in the text reflect 95% confidence intervals unless otherwise noted (mean ± CI). Bonferroni corrections were used to control for multiple comparisons.

## Results

Since there have been few studies examining shoulder reflexes elicited by translations of the glenohumeral joint, we begin by characterizing the nature of the elicited reflexes. We follow by providing a detailed evaluation of our hypotheses related to the stretch-evoked reflexes elicited in the rotator cuff muscles and primary shoulder movers. Finally, we describe the results of our control experiment comparing reflex characteristics recorded in the same muscle with surface and fine-wire intramuscular electrodes.

### Nature of the Stretch-Evoked Reflexes Elicited by Glenohumeral Translations

**Figure 3:**
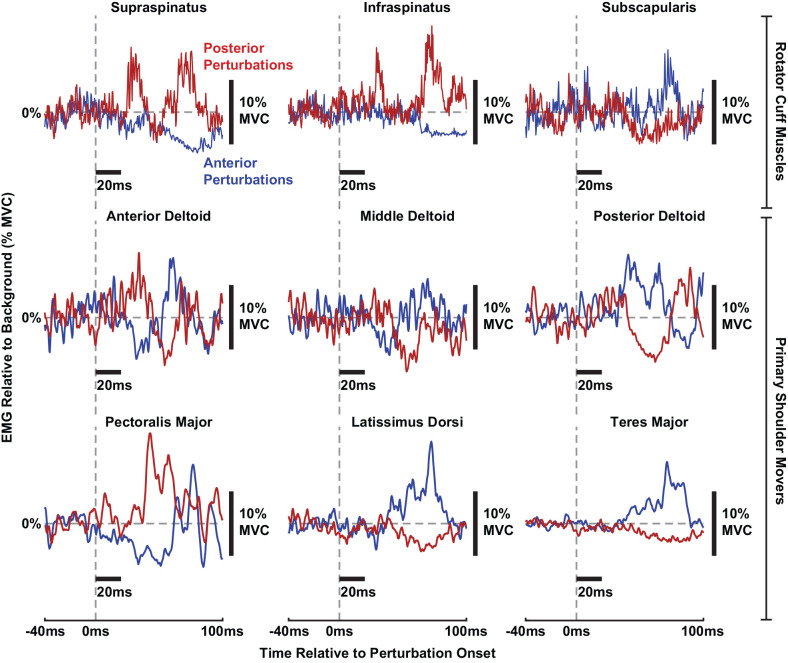
Individual stretch-evoked reflexes responding to anterior (blue) and posterior (red) perturbations in all muscles from a typical participant. All trials shown are from torque levels of 10% MVC in a torque direction that generated a large background activity for each muscle. All EMGs are displayed relative to background activity. Thick black bars are included to display the scale of the x-axes (20 ms per horizontal bar) and y-axes (10% MVC of ∆EMG per vertical bar), respectively.

Stretch-evoked reflexes were elicited by translations of the glenohumeral joint in all shoulder muscles recorded in our experiment. While no prior studies have described how shoulder muscles are stretched or shortened by translational perturbations, the expected length changes of the primary shoulder movers can be inferred from their anatomical orientation at the glenohumeral joint. We expect that muscles would be stretched by translations moving in a direction opposite of where they sit with respect to the glenohumeral joint, and the muscles would be shortened by translations moving in the same direction. For example, the *anterior deltoid* and *pectoralis major* sit anteriorly to the glenohumeral joint and are likely stretched by posterior perturbations. Accordingly, the *anterior deltoid* and *pectoralis major* displayed facilitatory reflexes in response to posterior perturbations and suppressive reflexes in response to anterior perturbations. In contrast, the *posterior deltoid*, *teres major*, and *latissimus dorsi* are oriented posteriorly to the glenohumeral joint and are likely stretched by anterior perturbations. Predictably, these three muscles displayed facilitatory reflexes in response to anterior perturbations and suppressive reflexes in response to posterior perturbations. Unlike the other primary shoulder movers, the *middle deltoid* is oriented superiorly to the glenohumeral joint. Reflexes in the *middle deltoid* did not consistently demonstrate facilitation or suppression responses to perturbations in either direction. It is more difficult to predict how translational perturbations would stretch or shorten the rotator cuff muscles given their compact anatomy around the glenohumeral joint. The *supraspinatus* and *infraspinatus* are roughly oriented posterosuperiorly and posteriorly, respectively, to the glenohumeral joint, yet both displayed facilitation in response to posterior perturbations and suppression in response to anterior perturbations. Likewise, the *subscapularis* is roughly oriented anteriorly to the joint but displayed facilitation in response to anterior perturbations and suppression in response to anterior perturbations. Overall, most facilitatory and suppressive reflexes were broad and monophasic, yet in some muscles such as the *supraspinatus*, *infraspinatus*, and *anterior deltoid*, brisk and biphasic facilitatory responses were observed. Examples of reflexes in each muscle can be observed for a typical participant in Figure 3.

### Reflex Amplitudes Were Larger in Rotator Cuff Muscles Than in Primary Shoulder Movers

**Figure 4:**
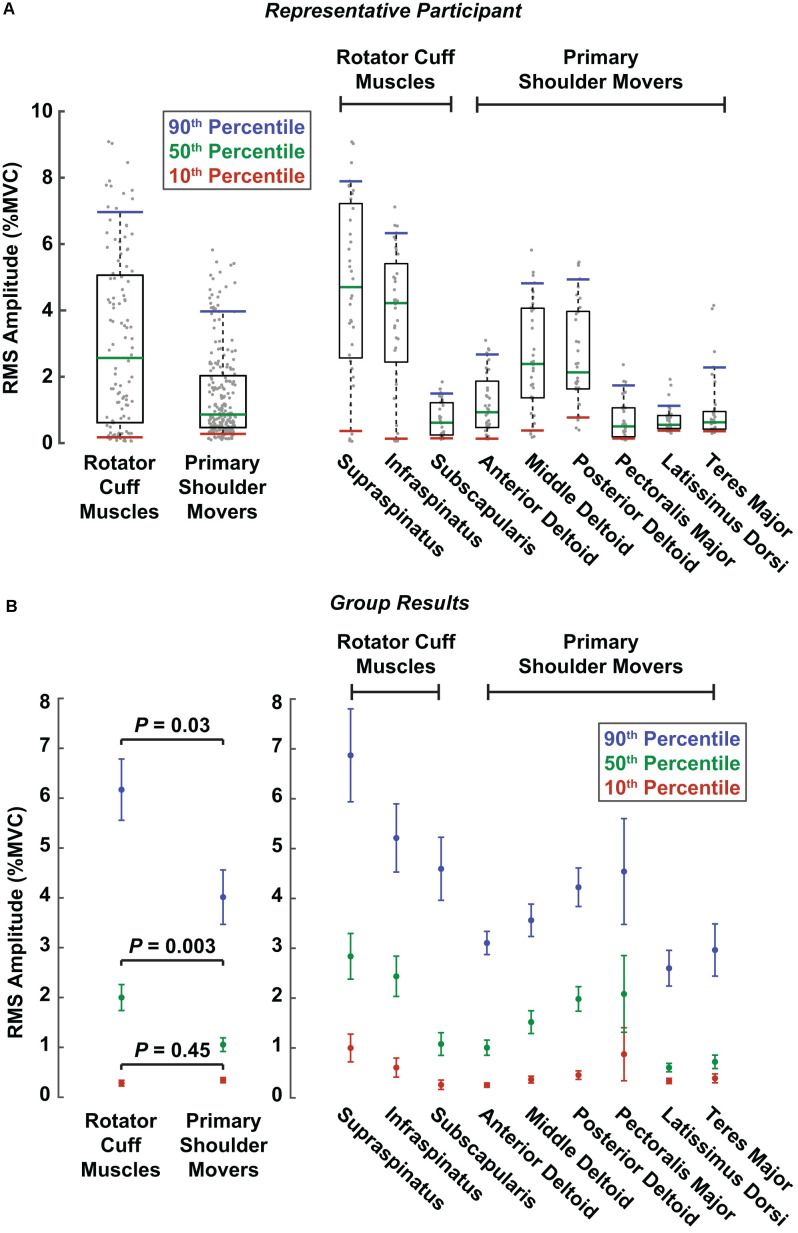
Reflex amplitudes comparing muscle groups and individual muscles. Reflex amplitudes were summarized as RMS amplitudes, which were calculated from the root mean square of the reflexes in each perturbation direction for an individual trial. Colored lines represent the RMS amplitudes corresponding to the 10th (red), 50th (green), and 90th (blue) percentiles across all trials at torque levels of 10% MVC. The three percentiles correspond approximately to the small, median, and large amplitudes across all trials, respectively, for a given muscle or muscle group. **(A)** RMS amplitudes are displayed for a representative participant. Gray dots represent RMS amplitudes from all the participant’s individual trials in both perturbation directions for a given muscle or muscle group. The boxes represent the interquartile range (25th and 75th percentiles), and the whiskers span from the 10th to 90th percentiles. **(B)** RMS amplitudes for all participants. Error bars represent the standard errors of the RMS amplitude estimates at each respective percentile.

Due to the multiphasic nature of the reflexes observed in several muscles, we used the RMS of the average rectified EMG between 20 and 100 ms to assess the aggregate change in muscle activity elicited by translational perturbations of the shoulder. We found that this measure of reflex activity, or the RMS amplitude, was larger in the rotator cuff muscles than in the primary shoulder movers. This was evaluated by comparing distributions of RMS amplitudes elicited as participants generated voluntary torques of 10% MVC, the most active condition in our experimental design. Distributions were created for each muscle, incorporating all six volitional torque directions and both perturbation directions. Results for a typical participant are shown in Figure 4A. The difference in RMS amplitude between muscles groups was driven by large reflexes in the *supraspinatus* and *infraspinatus* and small reflexes in the *latissimus dorsi* and *teres major*. Across all participants, the median RMS amplitude was nearly twice as large in the rotator cuff muscles (2.0 ± 0.5% MVC) compared to the primary shoulder movers (1.0 ± 0.3% MVC; *P* = 0.003; Figure 4B). Similar differences were observed for the large (90th percentile) reflexes elicited in each group. The large reflexes in the rotator cuff muscles (6.1 ± 1.2% MVC) were approximately 50% greater than those in the primary movers (4.0 ± 1.1% MVC; *P* = 0.03). In contrast, minimal differences were observed between the small (10th percentile) reflexes in each group, where reflexes in the primary movers (0.3 ± 0.1% MVC) were slightly larger than those in the rotator cuff (0.2 ± 0.1%; *P* = 0.45). The largest reflexes were observed in the *supraspinatus* (median: 2.8 ± 0.9% MVC; large: 6.9 ± 1.8% MVC) and *infraspinatus* (median: 2.4 ± 0.8% MVC; large: 5.1 ± 1.3% MVC; Figure 4B). The smallest were observed in the *latissimus dorsi* (50th percentile: 0.6 ± 0.2% MVC; 90th percentile: 2.6 ± 0.7% MVC) and *teres major* (50th percentile: 0.7 ± 0.3% MVC; 90th percentile: 2.9 ± 1.0% MVC; Figure 4B).

**Figure 5:**
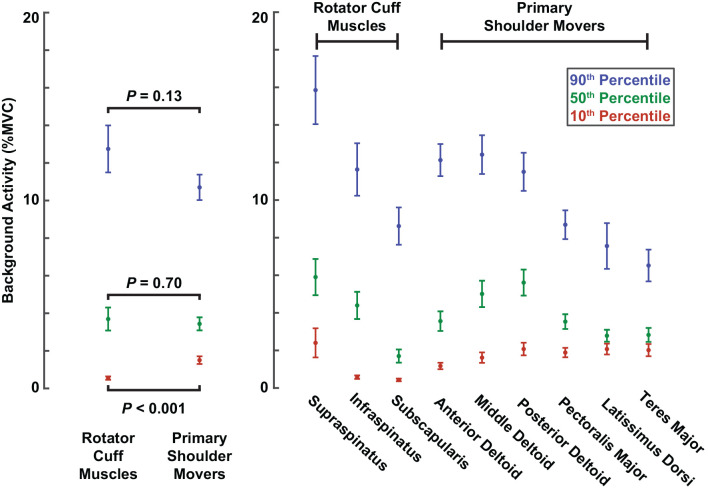
Background activity comparing muscle groups and individual muscles. Colored dots represent background activities corresponding to the 10th (red), 50th (green), and 90th (blue) percentiles across all trials with torque levels of 10% MVC. Error bars represent the standard errors of each estimate.

The larger reflexes observed in the rotator cuff muscles may have been influenced by differences in background muscle activity between the two muscle groups, given that reflex amplitudes typically scale with background activity. However, across all participants, the median background activity at torque levels of 10% MVC barely differed between rotator cuff muscles (3.7 ± 1.2% MVC) and primary shoulder movers (3.4 ± 0.7% MVC; *P* = 0.70; Figure 5). The large (90th percentile) background activity in rotator cuff muscles (12.8 ± 2.5% MVC) was larger than in the primary shoulder movers (10.7 ± 1.3% MVC; *P* = 0.13), but the difference was still relatively small. In contrast, the small (10th percentile) background activity was larger in the primary shoulder movers (1.5 ± 0.4% MVC) than the rotator cuff muscles (0.5 ± 0.2% MVC; *P* < 0.001). Therefore, factors beyond the background activity of each muscle likely contributed to the differences in reflex amplitude between muscle groups.

### Gain-Scaling Was More Prominent in Rotator Cuff Muscles Than in Primary Shoulder Movers

**Figure 6:**
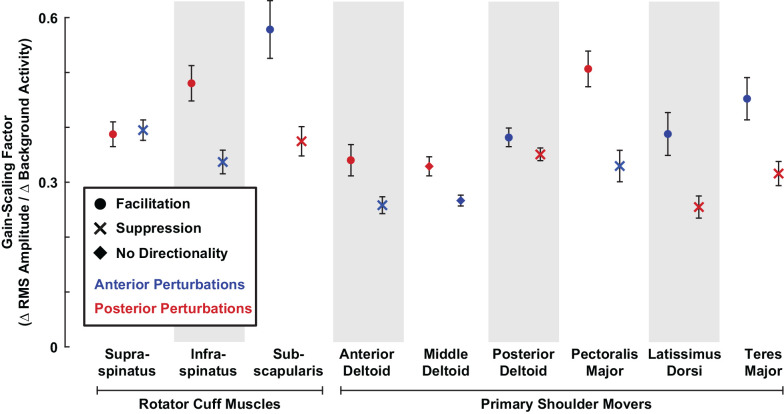
Gain-scaling in all shoulder muscles. The gain-scaling factor for each muscle was estimated from the relationship between the RMS amplitude and background activity in each muscle stratified by perturbation direction. Error bars represent the standard errors of the estimated gain-scaling factors.

To determine if differences in reflex amplitude reported above were due to differences in reflex sensitivity to background activity, we computed the gain-scaling factor for each muscle, which defines the change in RMS amplitude resulting from a change in background activity. The gain-scaling factor was significantly different from zero in all muscles (all *P* < 0.001). Our data were described well by a model predicting RMS amplitudes based only on background activity, muscle type, and perturbation direction as the independent fixed factors (*R^2^* = 0.89). The gain-scaling factors ranged from 0.25 to 0.55 (∆ RMS amplitude/∆ background activity) across all muscles and perturbation directions (Figure 6). For a given muscle, gain-scaling factors were nearly always larger in the perturbation direction that elicited facilitatory reflexes than the perturbation direction that elicited suppressive reflexes. The only exception was in the *supraspinatus*, which displayed similar gain-scaling in both perturbation directions.

Gain-scaling was larger in the rotator cuff muscles than in the primary shoulder movers (Figure 6). Gain-scaling factors in the rotator cuff muscles were 0.43 ± 0.04 (∆ RMS amplitude/∆ background activity) for anterior perturbations and 0.41 ± 0.03 for posterior perturbations. In contrast, the values for the primary shoulder movers were 0.35 ± 0.03 and 0.35 ± 0.03 for anterior and posterior perturbations, respectively. The difference between muscle groups had a statistical significance of *P* = 0.002 for both perturbation directions. On average across both perturbation directions, gain-scaling factors in the cuff muscles were 0.07 ± 0.03 larger than in the primary shoulder movers (*P* < 0.001). Intercepts in the model were small but significant for the rotator cuff muscles (0.20 ± 0.03% MVC, *P* < 0.001) and primary shoulder movers (−0.11 ± 0.02% MVC, *P* < 0.001); these also differed significantly (*P* < 0.001). The similar degree of average gain-scaling following anterior and posterior perturbations suggests that shoulder muscles collectively generate balanced resistance to external disturbances coming from each direction. Gain-scaling factors were also larger in rotator cuff muscles than in primary shoulder movers when stratifying by perturbation directions that elicited facilitatory (0.47 ± 0.04 vs. 0.42 ± 0.04; *P* = 0.008) or suppressive reflexes (0.36 ± 0.04 vs. 0.30 ± 0.02; *P* < 0.001).

**Figure 7:**
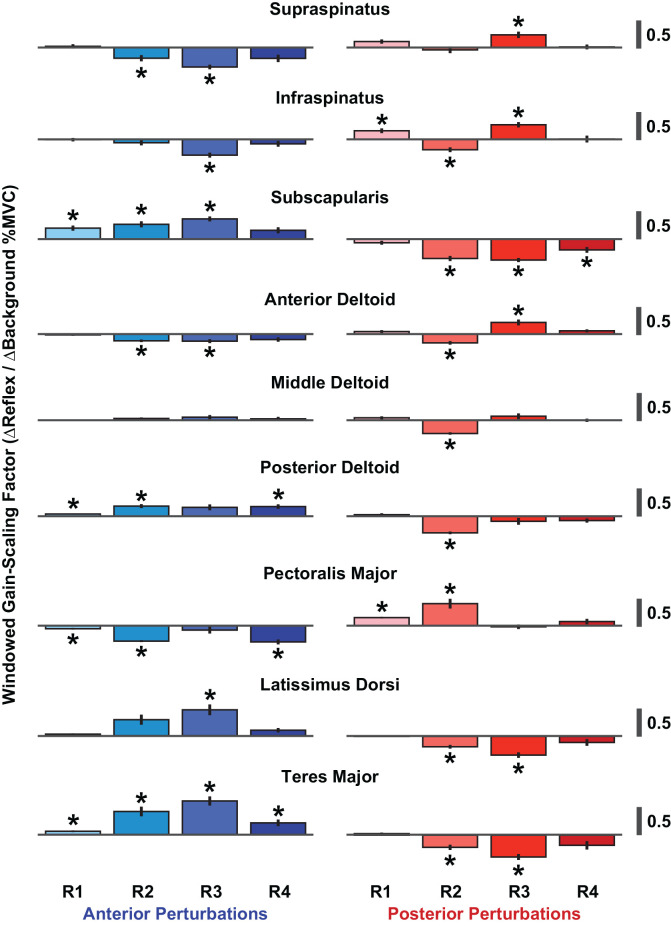
Windowed gain-scaling factors for stretch-evoked reflexes in shoulder muscles responding to translations of the glenohumeral joint. The gain-scaling factor was computed from the relationship between a muscle’s background activity and its reflex response in a given reflex window. Gain-scaling factors marked with an asterisk were significantly different from zero (*α* = 0.05/72). Vertical black lines represent the standard errors of the gain-scaling factors. Reflex windows: R1 (20–40 ms), R2 (40–60 ms), R3 (60–80 ms), R4 (80–100 ms).

The time-course of the reflexes elicited in each muscle was quantified by examining the average rectified EMG in four time windows following perturbation onset: R1 (20–40 ms), R2 (40–60 ms), R3 (60–80 ms), and R4 (80–100 ms). We assessed the gain-scaling in each window, as described above for the RMS reflex amplitudes. The largest gain-scaling was typically observed in R2 or R3 (Figure 7). These results were obtained by grouping data across all six volitional torque directions.

Given that previous studies have shown that reflexes in shoulder muscles are affected by the specific tasks being performed (Pruszynski et al., 2008; Krutky et al., 2010; Nicolozakes, 2021), we also assessed gain-scaling in each window separately for each of the six torque directions used in our protocol. We quantified the improvement in goodness-of-fit between the models that estimated gain-scaling with and without grouping data across all six torque directions. The more complicated model led to only a modest improvement in the fit accuracy (median ∆R^2^: +0.03, IQR: +0.02–0.06; Supplementary Figure 1). These findings suggest that the reflexes recorded in our study were most sensitive to changes in the background activity of the homonymous muscle rather than the coordinated activity of all muscles contributing to each of the tested torque directions. Interestingly, this is quite different than reflexes elicited by shoulder rotations (Nicolozakes, 2021).

### Comparisons of Reflex Latencies Between Rotator Cuff Muscles and Primary Shoulder Movers

**Figure 8:**
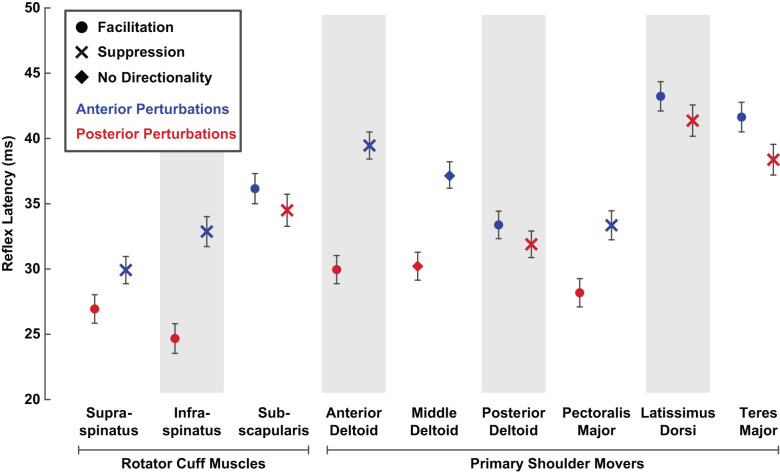
Average latencies of stretch-evoked reflexes responding to translational perturbations of the glenohumeral joint. Reflex latencies were estimated as the time after perturbation onset when the average rectified EMG diverged positively or negatively from background activity by at least two standard deviations. Latencies were estimated separately for each perturbation direction. Only latency data from active trials (5% or 10% MVC) that elicited a reflex exceeding the threshold were included. Error bars represent the standard errors of the reflex latencies.

In addition to comparing reflex amplitudes between rotator cuff muscles and primary shoulder movers, we also compared reflex latencies, which represent a secondary measure relevant to the efficacy of the reflex response. The reflex latencies in the rotator cuff muscles were shorter than those in the primary shoulder movers. Specifically, reflex latencies in rotator cuff muscles were 5 ± 1 ms shorter, on average, than the latencies of the primary shoulder movers for facilitatory responses (29 ± 2 ms vs. 34 ± 2 ms; *P* < 0.001). The shortest mean facilitatory latencies were observed in the* supraspinatus* (27 ± 2 ms) and the *infraspinatus* (25 ± 2 ms), and the longest in the *latissimus dorsi* (43 ± 2 ms) and *teres major* (42 ± 2 ms; Figure 8).

Facilitatory reflex latencies became shorter with increased background activity in the three muscles that had the slowest facilitatory responses: the *subscapularis*, *latissimus dorsi*, and *teres major*. This decrease may be due to muscle activation creating a more effective transmission of the applied glenohumeral translation to the proprioceptors mediating the reflex response, or to decreasing the threshold of the relevant motoneurons. In these three muscles, facilitatory reflex latencies decreased by approximately 1 ms for each percentage increase in background activity (*subscapularis* ∆: −0.9 ± 0.6 ms/%MVC, *P* = 0.003; *latissimus dorsi* ∆: −1.1 ± 0.7 ms/%MVC, *P* = 0.001; *teres major* (∆: −1.1 ± 0.8 ms/%MVC, *P* = 0.005). Smaller, nonsignificant changes in latency with increased muscle activation were observed in the other six muscles. Despite these activation-dependent changes, the differences in the facilitatory latencies were maintained at the highest activations tested.

Reflex latencies in rotator cuff muscles were also 5 ± 1 ms shorter than in primary shoulder movers for suppressive responses (32 ± 2 ms vs. 37 ± 2 ms; *P* < 0.001). A smaller range of latencies was observed for suppressive responses compared to facilitatory responses, with the shortest mean latency observed in the *supraspinatus* (30 ± 2 ms) and the longest in the *latissimus dorsi* (41 ± 2 ms). Within each muscle, reflexes elicited by posterior perturbations always occurred at a shorter latency than those elicited by anterior perturbations, regardless of the nature of the responses (facilitatory or suppressive; Figure 8). Such differences suggest a quicker overall response to posterior perturbations. In all nine muscles, only small, nonsignificant relationships between latency and muscle activation were observed for suppressive responses.

### Recording Modality Influenced Measured Reflex Latencies

**Figure 9:**
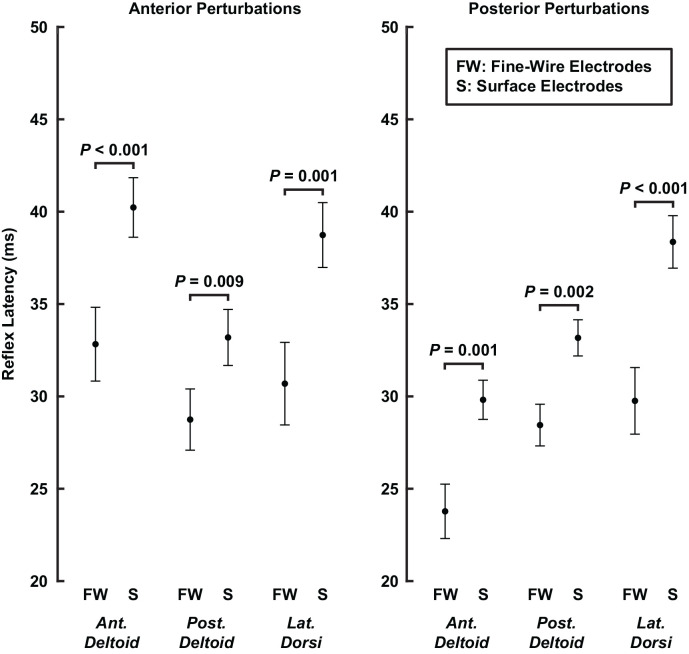
Average latencies of stretch-evoked reflexes recorded in the same muscle with fine-wire intramuscular electrodes and surface electrodes. Latencies were estimated separately for each perturbation direction. Error bars represent the standard errors of the reflex latencies.

In our control experiment, we compared reflex latencies, amplitudes, and gain-scaling factors between reflexes recorded in the same muscle with surface or fine-wire electrodes. Across the three muscles, reflex latencies were on average 7 ± 2 ms shorter in reflexes recorded with fine-wire electrodes than those recorded with surface electrodes (*P* < 0.001; Figure 9). The differences were most pronounced in the *latissimus dorsi* (anterior: 8 ± 5 ms shorter, *P* = 0.001; posterior: 9 ± 4 ms shorter, *P* < 0.001) and least pronounced in the *posterior deltoid* (anterior: 4 ± 3 ms shorter, *P* = 0.009; posterior: 5 ± 3 ms shorter, *P* = 0.002). Differences in electrode type used to record reflexes from the rotator cuff muscles and primary shoulder movers may therefore have contributed to the differences in reflex latency recorded in our primary experiment.

Reflex amplitudes were impacted less by electrode type than were the reflex latencies. We found that our measures of reflex amplitude differed slightly or not at all between surface and fine-wire electrodes. Median RMS amplitudes (50th percentile) on average across muscles did not differ between electrode type (∆ fine-wire — surface = −0.05 ± 0.39% MVC; *P* = 0.81). Similar but slightly larger results were observed for the 90th percentile (∆ = 0.6 ± 0.7% MVC, *P* = 0.14), which were larger when recorded with fine-wire electrodes. This statistically insignificant bias across all muscles was due to small but significant increases in the anterior deltoid (∆ = 1.1 ± 0.5% MVC, *P* < 0.001) and latissimus dorsi (∆ = 0.5 ± 0.5% MVC, *P* = 0.03) amplitudes when using fine-wire electrodes. For smaller reflexes (10th percentile), amplitudes again did not differ between electrode type (∆ = −0.02 ± 0.20, *P* = 0.87). This difference only reached significance for the anterior deltoid (∆ = −0.11 ± 0.04% MVC, *P* < 0.001), which had larger amplitudes when recorded with surface electrodes. The average differences in gain-scaling between electrode type (anterior perturbations: *∆* = 0.02 ± 0.12 ∆ RMS amplitude/∆ background activity, *P* = 0.76; posterior perturbations: *∆* = 0.05 ± 0.04, *P* = 0.06) were also less than we observed in our primary experiments. These results suggest that differences in electrode type may have influenced the magnitude of the amplitude effects reported in our primary experiment but not the overall conclusions.

## Discussion

The purpose of this study was to compare stretch-evoked reflexes elicited by translations of the glenohumeral joint between rotator cuff muscles and muscles that are primary movers of the shoulder. Stretch-evoked reflexes were elicited in all muscles we studied. Reflex amplitudes were larger in the rotator cuff muscles than in the primary shoulder movers, with the highest amplitudes observed in the *supraspinatus* and *infraspinatus*. The increased amplitudes in these rotator cuff muscles were due to a larger level of background activity and an increased scaling with background activity, quantified by a gain-scaling factor for each muscle. Additionally, reflex latencies were shorter in rotator cuff muscles than in primary shoulder movers, but the differences observed may have been influenced by recording EMG with different types of electrodes. Our findings demonstrate that translations of the glenohumeral joint elicit strong stretch-evoked reflexes and that these reflexes are most vigorous in the rotator cuff muscles thought to be essential for shoulder stability. These involuntary responses likely arise from the diversity of proprioceptors within the muscles and passive structures surrounding the shoulder. Their actions serve to amplify the stabilizing properties of the rotator cuff muscles that have already been identified during volitional control, providing a brisk response to glenohumeral translations that should promote centering the humeral head within the glenoid fossa.

### Factors Contributing to Different Reflex Amplitudes Between Shoulder Muscles

The shoulder is embedded with many proprioceptors that could have contributed to the reflexes observed in this study. While our study was not designed to identify the specific sensory organs contributing to reflexes elicited by glenohumeral translations, it is insightful to consider the potential sources of afferent information.

Muscle spindles are commonly assumed to be a primary source of afferent information when studying stretch-evoked reflexes. While we assume that they contributed to the responses we observed, it is unlikely that spindles alone accounted for the differences between the rotator cuff muscles and the primary movers of the shoulder. Muscle spindles are sensitive to changes in muscle length and its derivatives (Poppele and Bowman, 1970; Finley et al., 2013; Blum et al., 2020), and the amplitude of the elicited reflex increases with increasing changes in muscle length (Nichols and Houk, 1976; Neilson and Mccaughey, 1981; Cathers et al., 1999). The largest changes in muscle length for our study would be expected in muscles with lines of action that are most closely aligned to the anterior or posterior perturbations used to elicit reflexes, which are the primary shoulder movers (Ackland and Pandy, 2009). Contrary to what would be expected if the elicited reflexes were due solely to changes in muscle length, we observed that the muscles with lines of action nearly orthogonal to the applied perturbations, the *supraspinatus* and *infraspinatus* (Ackland and Pandy, 2009), had the largest reflex amplitudes. These larger amplitudes were not due simply to increased background activity in the rotator cuff muscles but also to an increased sensitivity of the elicited reflexes, which we quantified by the gain-scaling of the response in each muscle (Matthews, 1986). Hence, proprioceptors other than muscle spindles are likely to have contributed to the enhanced reflexes we observed in the rotator cuff muscles, though we cannot rule out differences in spindle density across muscle groups crossing the shoulder or differences in muscle-tendon compliance.

Additional proprioceptors that could be relevant to reflex activation of the shoulder musculature include the free nerve endings, Ruffini corpuscles, Golgi tendon organs, and Pacinian corpuscles within the glenohumeral capsule, its constituent ligaments, and the glenoid labrum (Bresch and Nuber, 1995; Vangsness et al., 1995; Gohlke et al., 1998; Guanche et al., 1999; Steinbeck et al., 2003; Witherspoon et al., 2014). These structures would be strained by translational perturbations of the glenohumeral joint (Brenneke et al., 2000), and the afferents within them have been shown to elicit strong reflexes in shoulder muscles that are most consistently observed in the rotator cuff (Voigt et al., 1998). Similar findings have been observed for stimulation of afferents from the coracoacromial ligament (Diederichsen et al., 2004) which, though external to the capsule, provides evidence for the broad innervation of passive structures within the shoulder. The reflexes we observed likely integrated afferent information from these passive structures along with those originating from muscle spindles, which may have contributed to the larger amplitudes observed in the rotator cuff muscles relative to those in the primary movers. Integration of afferent information from capsular proprioceptors would likely be much larger for reflexes elicited by glenohumeral translations compared to those elicited by glenohumeral rotations given that the latter should generate less strain on the passive structures. Interestingly, both the Voigt et al. (1998) and Diederichsen et al. (2004) studies noted strong suppressive responses in the shoulder muscles upon stimulation of the capsular and ligamentous afferents. In contrast, the translational perturbations used in our study elicited facilitative and suppressive reflexes. This difference may also result from the integrated effects of different sensory organs and pathways that are stimulated when the intact shoulder is translated as opposed to the more focused electrical stimulation used in prior work.

### Factors Contributing to Different Reflex Latencies Between Shoulder Muscles

We found that reflex latencies were approximately 5 ms shorter in the rotator cuff muscles than in the primary shoulder movers. These findings were driven in part by long latencies in the *latissimus dorsi* and *teres major* and short latencies in the *supraspinatus* and *infraspinous*. However, in our control experiment, reflexes were approximately 7 ms shorter when recorded with fine-wire electrodes than with surface electrodes. While these instrumentation differences do not account for the full range of reflex latencies we observed (Figure 8), we cannot rule out the possibility that they are large enough to explain the average differences between rotator cuff muscles and primary movers. Interestingly, Day et al. (2012) also found reflexes in the rotator cuff muscles are faster than in the primary movers following unexpected internal and external rotation perturbations to the shoulder. They reported the fastest responses in the *infraspinatus* and *subscapularis*, and slower responses in the *anterior* and *posterior deltoid*. Notably, they also used fine-wire electrodes and surface electrodes to record reflexes in the rotator cuff muscles and primary shoulder movers, respectively, which may have contributed to their findings. A more careful assessment of any possible latency differences between these groups of muscles will require the consistent use of fine-wire electrodes.

The two muscles with the longest latencies in our study also demonstrated the largest negative correlation between muscle activation and reflex latency. The decrease in reflex latencies with increased muscle activity could arise from a more effective transmission of the perturbation to the muscles, a decreased threshold of the motoneuron pool, or increased spindle sensitivity arising from gamma activation (Vallbo, 1974). Similar to our activation-dependent measures, Myers et al. (2003) found that the latencies of reflexes in the *latissimus* *dorsi*, elicited by glenohumeral rotations in healthy shoulders, are also longer than in other shoulder muscles during relaxed conditions and decrease with increasing muscle activation; above 20% MVC, the latencies of the *latissimus dorsi* were comparable to those in other shoulder muscles. While the latencies of the *latissimus dorsi* and *teres major* in our study still were larger than most other shoulder muscles at higher activations, we did not approach the levels of activation tested by Myers et al. (2003). Higher activations may have led to the same result.

We are aware of only one other assessment of reflex latencies occurring from translational perturbations of the humeral head. Latimer et al. (1998) applied anterior translational forces to the humeral head using a pulley system that dropped weights onto an outstretched arm. Reflex latencies were recorded under passive conditions in multiple rotator cuff muscles and primary shoulder movers. Across all the tested shoulder muscles, they reported latencies of passive reflexes that ranged from 110 to 220 ms. The passive conditions of that study likely increased reflex times due to poor mechanical transmission along with the neural factors described above. Importantly, voluntary responses to perturbations can occur with latencies as short as 100 ms (Hammond, 1956; Honeycutt and Perreault, 2012; Forgaard et al., 2015), so it is unclear if the latencies reported by Latimer et al. (1998) represent reflex or volitional responses.

Across all muscles, reflexes in our study occurred at average latencies of 25–45 ms. While the fastest responses are similar in latency to stretch-evoked reflexes elicited by rotational perturbations of the shoulder (Perreault et al., 2008; Muraoka and Kurtzer, 2020; Nicolozakes, 2021), most are slower than would be expected for monosynaptic stretch-evoked reflexes. These longer latencies suggest that the reflexes elicited by translational perturbations may arise from structures other than muscle spindles, as discussed above. While the source of the reflex responses was not addressed in our work, it is interesting to note that reflexes elicited by electrical stimulation of the glenohumeral capsule have been measured to have latencies of approximately 33 ms, which are more consistent with the latencies observed in our study (Voigt et al., 1998). These slower responses provide further evidence that secondary afferents and the sensors they innervate likely contributed to the net reflexes observed in this study.

### Methodological Considerations

Our study is among the first to assess reflexes elicited by translations of the glenohumeral joint, which can lead to dislocation when large enough to move the humeral head out of the glenoid fossa. These translations of the intact joint have the benefit of exciting all sensors that respond to joint translations during normal activities, and therefore could be considered more functional than the elegant mechanistic studies that have stimulated isolated elements within the sensory system of the shoulder. What is lost is the ability to identify the role of specific sensory systems in the net reflex responses we quantified. Modeling studies or more detailed experimental measures may help to bridge this gap.

In our control experiment, we found that reflex latencies were shorter when recorded with fine-wire intramuscular electrodes than when recorded with surface electrodes in the same muscle. These differences may have contributed to the shorter latencies we observed in the rotator cuff muscles since their anatomy, unlike the primary shoulder movers, required the use of fine-wire electrodes. Our results differ from those of prior comparisons of reflex latencies between surface and fine-wire electrodes, which found no differences between the two modalities (Wittek et al., 2001), although the standard deviations of their measurements were much higher than ours. Reflex amplitudes and gain-scaling factors were also slightly larger when recorded with fine-wire intramuscular electrodes. However, the differences were smaller than the differences we observed between rotator cuff muscles and primary shoulder movers in our primary experiment. Hence, these recording differences may have influenced the magnitude of the differences in reflex amplitude and gain-scaling we observed between muscle groups but not our overall conclusions. While studies analyzing shoulder muscle activity commonly record EMG with surface and fine-wire electrodes (Barden et al., 2005; Kibler et al., 2007; Day et al., 2012; Thomas et al., 2013), our results suggest that more consistent recording with fine-wire electrodes is warranted when the timing of EMG responses is of interest.

Our experiments were designed to assess shoulder reflexes elicited by glenohumeral translations. However, it was not possible to isolate our experimental perturbations solely to the glenohumeral joint, which would require a more direct interface with the bones of the humerus and scapula. We therefore cannot rule out small rotations occurring at the glenohumeral joint during the trials. We minimized soft tissue displacement by applying perturbations through a tight-fitting cast that interfaced with bony prominences on the humerus and by externally clamping the scapula. The comprehensive casting minimized glenohumeral rotations so that translations applied at the middle of the humerus were transmitted to the humeral head with minimal rotation of the humerus. This setup allowed us to use small, safe perturbations that avoided the possibility of dislocation, while still creating controlled translational strains at the glenohumeral joint.

It is also important to note that our results are limited to the posture we studied. We made all measurements with the shoulder oriented in 90° abduction, neutral rotation, and 20° horizontal flexion. Given that the tension of the glenohumeral capsule and the muscles’ lines of action are unique to a shoulder’s orientation (Turkel et al., 1981; Ackland and Pandy, 2009), reflexes are likely to vary at different shoulder postures. One example is the apprehension posture, which is linked to symptom reproduction in individuals with shoulder instability (Rowe and Zarins, 1981). We previously reported that the translational stiffness of the glenohumeral joint differs between the posture tested in our study and the apprehension posture (Nicolozakes et al., 2021). It is likely that reflexes also vary with posture due to differences in how shoulder muscles and passive glenohumeral structures are oriented in each posture (Turkel et al., 1981; Ackland and Pandy, 2009).

## Conclusions

In summary, we found that stretch-evoked reflexes elicited by glenohumeral translations were larger in rotator cuff muscles than reflexes in primary shoulder movers. The strong reflexes elicited in the rotator cuff muscles, whose amplitudes ranged from 33% to 55% of background activity, could play a substantial role in maintaining shoulder stability, since the actions of the rotator cuff increase shoulder stability by pulling the humeral head into the glenoid fossa. While strong reflex activation of the rotator cuff muscles seems to be an appropriate response to mitigate the effects of unexpected translations, the mechanisms driving this response remain unclear since the muscles that have the largest reflex response to translations would also have relatively small length changes. This suggests a coordinated sensory response that integrates information from multiple structures within the shoulder, rather than only the muscle spindles commonly associated with stretch-evoked reflexes. Injury to the glenohumeral capsule that occurs following dislocation reduces shoulder proprioception and impacts reflexes elicited by joint rotations (Lephart et al., 1994; Zuckerman et al., 2003; Myers et al., 2004); what remains to be seen is if these disruptions also alter the reflexively elicited protective responses observed in this study. If so, our results can serve as a valuable benchmark to compare the translational reflexes present in healthy shoulders to those altered in individuals who have suffered dislocations or other injuries that alter the passive and active structures contributing to shoulder stability.

## Data Availability Statement

The raw data supporting the conclusions of this article will be made available by the authors upon request, without undue reservation.

## Ethics Statement

The studies involving human participants were reviewed and approved by Northwestern University Institutional Review Board. The participants provided their written informed consent to participate in this study.

## Author Contributions

CN conceived the project, designed and performed the experiments, performed the analyses, and wrote the manuscript. MC-T performed the experiments and performed the analyses. DL designed the experiments and performed the analyses. AS and EP supervised the design of experiments and analyses of results. All authors contributed to the article and edited, revised, and approved the final submitted version of the manuscript.

## Conflict of Interest

The authors declare that the research was conducted in the absence of any commercial or financial relationships that could be construed as a potential conflict of interest.

## Publisher’s Note

All claims expressed in this article are solely those of the authors and do not necessarily represent those of their affiliated organizations, or those of the publisher, the editors and the reviewers. Any product that may be evaluated in this article, or claim that may be made by its manufacturer, is not guaranteed or endorsed by the publisher.
