# Supplementary material for: Translations of the Humeral Head Elicit Reflexes in Rotator Cuff Muscles That Are Larger Than Those in the Primary Shoulder Movers
Source: Front Integr Neurosci. 2022 Feb 2;15:796472. doi: 10.3389/fnint.2021.796472 (PMC8847177; doi:10.3389/fnint.2021.796472)
Supplement: Supplementary file 1 [file Data_Sheet_1.pdf]

frontiers

Supplementary Material

1 Supplementary Data

Reflexes in shoulder muscles elicited by rotations of the joint are affected by the specific tasks being performed (Pruszynski et al., 2008;Krutky et al., 2010). This can include the modulation of gain-scaling, or the relationship between the background activity of the homonymous muscle and its reflex amplitude, that depends on the coordinated activity of surrounding shoulder muscles (Nicolozakes, 2021).

We evaluated if there was any evidence of task-dependent gain-scaling in our present experiment. Specifically, we evaluated if the relationship between the background activity in a muscle and the reflex elicited within it changed depending on the direction of torque that subjects were producing voluntarily. This was accomplished by computing gain-scaling in two ways. First, we used a simple linear mixed-effects model as presented in the main manuscript. This modeled the relationship between a muscle's background activity and its reflex amplitude without considering the direction of voluntary torque. This simple model was compared to a more complex linear mixed effects model that computed a separate gain-scaling factor for each of the six torque directions. Separate gain-scaling factors were computed for each of the four reflex windows considered in our main study: R1 (20-40 ms), R2 (40-60 ms), R3 (60-80 ms), R4 (80-100 ms). We quantified the improvement in goodness-of-fit between the models that estimated gain-scaling with and without grouping data across all six torque directions.

As noted in our Results section, the more complicated model led to only a modest improvement in the fit accuracy (median ΔR^2: +0.03, IQR: +0.02-0.06). The improvements noted for each muscle and reflex window can be observed in **Supplementary Figure 1**.

2 Supplementary Figures and Tables

2.1 Supplementary Figures

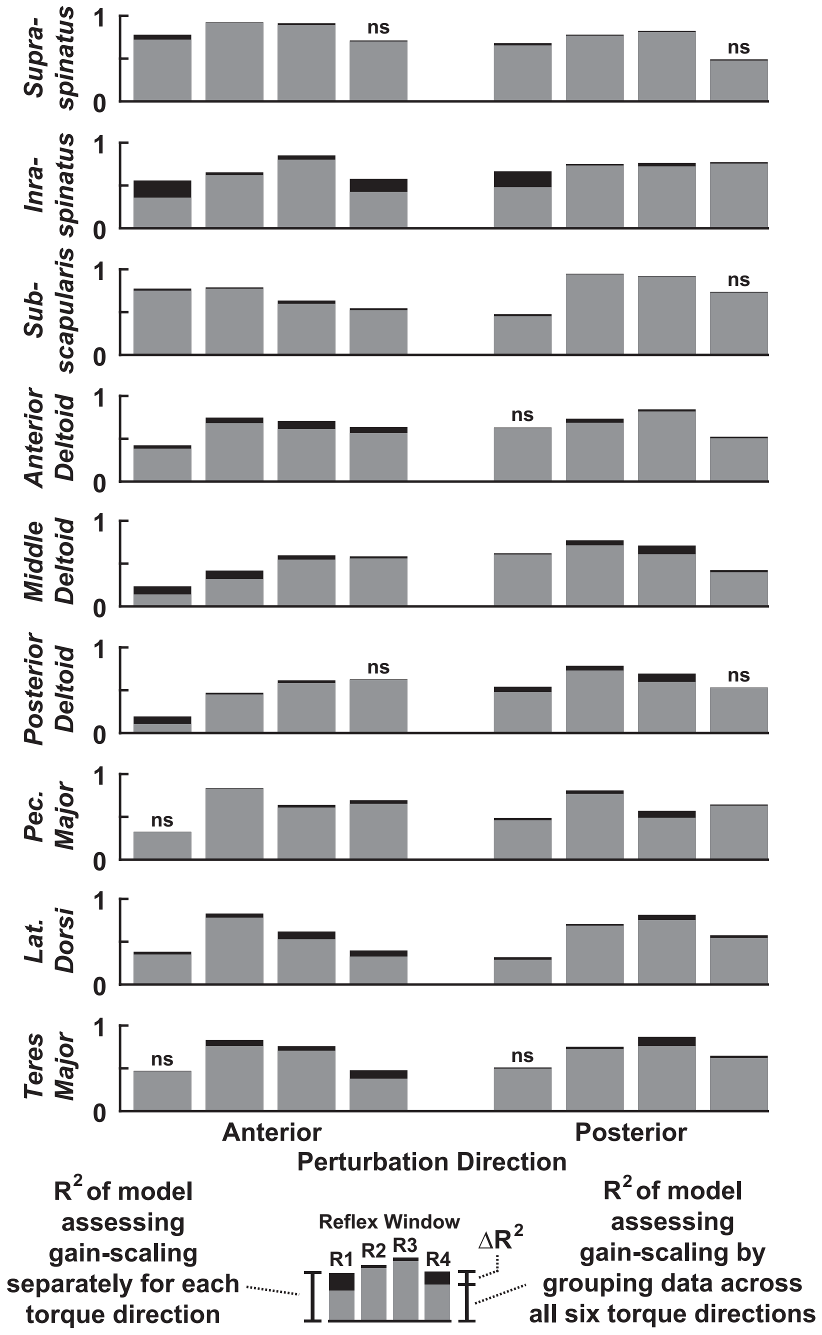

Supplementary Figure 1. Improvements in goodness-of-fit (R^2) between models estimating gain-scaling factors with and without grouping data across torque directions. Each layered gray and black bar represents the R^2 for the two models in the same condition (muscle, perturbation direction, and reflex window). All models that assessed gain-scaling separately for each torque direction were significantly improved compared to the corresponding model grouping data across torque directions unless noted as not significant (ns).

3 References

Krutky, M.A., Ravichandran, V.J., Trumbower, R.D., and Perreault, E.J. (2010). Interactions between limb and environmental mechanics influence stretch reflex sensitivity in the human arm. *J Neurophysiol* 103**,** 429-440.

Nicolozakes, C.P. (2021). *Quantifying the biomechanical and neural factors contributing to translational shoulder stability.* PhD Thesis, Northwestern University.

Pruszynski, J.A., Kurtzer, I., and Scott, S.H. (2008). Rapid motor responses are appropriately tuned to the metrics of a visuospatial task. *J Neurophysiol* 100**,** 224-238.
